# News and Notes

**Published:** 1909-01

**Authors:** 


### NEWS AND NOTES

Dr. Chas. Boynton is in New York for several days.

Dr. Amster, of Atlanta is still in New York City.

Dr. and Mrs. James R. Garner announce the birth of a son.

Dr. Eugene Murphy, of Augusta, attended the automobile races at Savannah.

Dr. W. T. Bull still continues critically ill at his apartments, sixteenth floor of the Plaza Hitel, New York.

Dr. and Mrs. Pattillo have returned from their bridal tour and are at home to their friends at Elizabeth street.

The Tri State Medical Association convened at Memphis, Tenn., November 24th. Several very interesting papers were read.

Dr. Wyman W. Pilcher, of Warrenton, Ga., was in Atlanta a few days since. Dr. Pilcher brought a case to the State Pasteur Institute for treatment.

Dr. W. S. Goldsmith, of Atlanta was one of the speakers on the first day at the twenty-first annual meeting of the Southern Surgical Association, in St. Louis, December 16th.

Dr. Smith Ely Jelliffe has retired from the co-editorship of the New York Medical Journal. Dr. Frank P. Foster remains, as heretofore, the editor-in-chief.

The International Typographical Union have appropriated $10,000 to establish a tent sanitorium for the tuberculous in Arizona.

---

Dr. Armstrong, Dr. Jas. H. Crawford, Dr. John S. Hurt and Dr. Willis Jones attended the automobile races at Savannah, November 25th to 26th. All report a delightful time and royal treatment by members of the profession in that hospitable city.

---

At the regular meeting of the Fulton County Medical Society, December the 17th, the following officers were elected for the ensuing year: President, Dr. Cyrus W. Strickler; vice-president, Dr. J. Ross Simpson; treasurer, Dr. A. H. Lindorme; secretary, Dr. Edgar G. Ballenger; censor, Dr. Michael Hoke.

---

It is with deep regret that we note the death of Dr. Andrew J. McCosh, of New York, Professor of Clinical Surgery in the medical department of Columbia University and visiting surgeon to the Presbyterian Hospital. The profession has suffered a distinct loss in the death of Dr. Cosh.

Dr. John P. Houston, one of the most prominent physicians of East Alabama, was struck by a train while driving. He received injuries from which he later died at his home in Arabecoochee.

---

### DEATH OF DR. C. C. STOCKARD.

It is with deep regret that we note the death of Dr. C. C. Stockard, a well known and highly esteemed physician of Atlanta.

Dr. Stockard died January 1, after a very sudden and severe illness, at the age of 55. He was a graduate of the University of Nashville and afterwards studied in Vienua. In 1892 he came to Atlanta, where he has been connected with several sanatariums, making a special study of drug addictions. The remains were taken to his former home, Columbus, Miss.

